# Phase Ib study of PRT543, an oral protein arginine methyltransferase 5 (PRMT5) inhibitor, in patients with advanced splicing factor-mutant myeloid malignancies

**DOI:** 10.1038/s41375-025-02515-8

**Published:** 2025-01-24

**Authors:** Jan Philipp Bewersdorf, Xiaoli Mi, Bin Lu, Andrew Kuykendall, David Sallman, Manish Patel, Don Stevens, Alexander Philipovskiy, Grerk Sutamtewagul, Lucia Masarova, Gina Keiffer, Amit Verma, Neha Bhagwat, Min Wang, Andrew Moore, Joseph Rager, Diane Heiser, Sunhee Ro, Wan-Jen Hong, Omar Abdel-Wahab, Eytan M. Stein

**Affiliations:** 1 Department of Medicine; Leukemia Service, Memorial Sloan Kettering Cancer Center, New York, NY USA; 2 Yale University and Yale Cancer Center, New Haven, CT USA; 3 Moffitt Cancer Center, Tampa, FL USA; 4 Florida Cancer Specialists, Sarasota, FL USA; 5 Norton Cancer Institute, Louisville, KY USA; 6 Florida Cancer Specialists, Lake Mary, FL USA; 7 University of Iowa, Iowa City, IA USA; 8 MD Anderson Cancer Center, Houston, TX USA; 9 Department of Medical Oncology, Sidney Kimmel Cancer Center, Thomas Jefferson University Hospital, Philadelphia, PA USA; 10 Montefiore Einstein Comprehensive Cancer, Bronx, NY USA; 11 Prelude Therapeutics, Wilmington, DE USA; 12 Molecular Pharmacology Program, Sloan Kettering Institute, Memorial Sloan Kettering Cancer Center, New York, NY USA

**Keywords:** Targeted therapies, Myelodysplastic syndrome

## Abstract

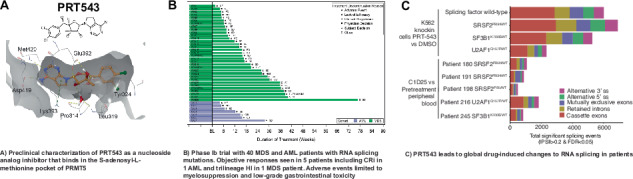

Mutations in RNA splicing factor genes are common in patients with myelodysplastic neoplasms (MDS) and secondary acute myeloid leukemia (AML) [1–3]. Cells bearing these mutations have been shown to be preferentially sensitive to pharmacologic modulation of splicing compared to wild-type (WT) cells [4, 5].

Protein arginine methyltransferase 5 (PRMT5) is an arginine methyltransferase which utilizes the methyl donor S-adenosylmethionine (SAM) to symmetrically methylate arginine residues on a variety of proteins [6, 7]. PRMT5-dependent symmetric di-methyl arginine (SDMA) mark is essential for spliceosome assembly and normal splicing [8]. Several preclinical studies have demonstrated that PRMT5 inhibition results in synthetic lethality with splicing factor mutant leukemia cells [1, 8]. Here we describe the discovery and characterization of PRT543, an oral potent, selective inhibitor of PRMT5, in preclinical studies as well as a phase I dose-escalation/expansion trial in patients with relapsed/refractory (R/R) myeloid neoplasms (NCT03886831).

This was a multicenter, open-label, sequential cohort, dose-escalation/expansion phase I study of PRT543 monotherapy in patients with advanced solid and hematologic malignancies. Only data from patients with AML, MDS, and MDS/MPN (myeloproliferative neoplasm) overlap are reported here. Data from the dose-escalation part leading to the identification of the recommended phase 2 dose (RP2D) of PRT543 35 mg daily 5x/week have been presented previously [9, 10]. Full details on study design are in Supplementary Methods. The primary endpoint of the dose escalation phase was to characterize dose-limiting toxicities (DLTs) and determine the RP2D of PRT543 monotherapy. In the dose expansion phase, the primary endpoint was best overall response rate (ORR) during first three response assessments. ORR was defined as a composite of complete remission (CR), partial remission (PR), marrow CR (mCR), and hematologic improvement (HI) per International Working Group (IWG) 2006 criteria for MDS and MDS/MPN patients and as a composite of CR, CR with incomplete hematologic recovery (CRi), PR or morphologic leukemia-free state (MLFS) per ELN 2017 response criteria for AML patients, respectively [11, 12]. Response assessment with bone marrow biopsies was performed at end of cycle 1 and every 2 cycles thereafter. All patients provided written informed consent prior to enrollment in the study. The study protocol was approved by the institutional review board at each participating site.

During dose-escalation patients were enrolled in a 3 + 3 design followed by a dose confirmation until the RP2D was achieved. Sample size justification is provided in Supplementary Methods. All patients who received any study drug were evaluated for safety and efficacy. Patients who received any study drug and were not evaluable for response were considered non-responders for efficacy analysis. Adverse event (AE) incidence was based on number of patients per AE category. Patient with multiple AEs classified to the same category were tabulated under worst toxicity grade for that AE category.

Full details on mutational analysis, xenograft studies, OncoPanel™ cell proliferation assay, RNA sequencing, PRMT5:MEP50 purification and crystallization, structure determination and functional characteristics are provided in the Supplementary Methods.

PRT543 is a nucleoside analog inhibitor that binds the SAM pocket of PRMT5. We solved the co-crystal structure of the PRMT5/MEP50 complex with PRT543 at resolution of 2.5 Å (Supplementary Fig. 1A) [13]. The dichloride phenyl ring of PRT543 displaces F327 and forms a unique π-π interaction with Y324 in the α1 helix, which is specific for PRMT5. As a result, PRT543 is highly selective against a wide array of 37 methyltransferases (Supplementary Fig. 1B, Supplementary Table 2). PRT543 potently reduced the methyltransferase activity of the PRMT5/MEP50 complex in vitro (Supplementary Fig. 1C). PRT543 was a SAM- and MTA-competitive and substrate-uncompetitive inhibitor of PRMT5 (Supplementary Fig. 1D), with a slow off rate and a long residence time indicative of a slow binding inhibitor (Supplementary Fig. 1E). Treatment with PRT543 showed a dose-dependent reduction of symmetrically dimethylated SmD3, a core splicing factor and PRMT5 substrate (Supplementary Fig. 1F). PRT543 robustly inhibited cell proliferation in a broad panel of cancer cell lines (Supplementary Fig. 1G) and tumor growth in AML xenograft models (Supplementary Fig. 2B).

Forty patients (18 lower-risk MDS, 11 higher-risk MDS, 7 AML, and 4 MDS/MPN overlap) were enrolled. Supplementary Table 4 and 5 provide an overview of patient and disease characteristics by disease type and enrollment to dose-escalation and -expansion phase, respectively. The most common splicing factor mutations among enrolled patients were *SF3B1, U2AF1*, and *SRSF2* in 13 (32.5%), 12 (30.0%), and 8 (20.0%) patients, respectively. Supplementary Fig. 3 provides each patient’s baseline genetic features, disease, and clinical response.

Preliminary results of the dose-escalation phase that included 11 patients with R/R MDS were presented previously [9]. MDS patients enrolled in the dose-escalation phase are included in the safety and efficacy analysis presented here. Most patients (*n* = 34; 85.0%) experienced ≥grade 3 treatment-emergent AEs. Anemia (*n* = 20; 50.0%) and thrombocytopenia (*n* = 12; 30.0%) as well as nausea (*n* = 11; 27.5%) and diarrhea (*n* = 11; 27.5%) were the most common treatment-emergent AEs of any grade. Table 1 and Supplementary Table 6 provide an overview of all any grade and treatment-related AEs. AEs leading to treatment interruption, dose reduction, and treatment discontinuation occurred in 16 (40.0%), 5 (12.5%), and 8 patients (20.0%), respectively. Serious AEs were documented in 13 patients (32.5%) and all unrelated to PRT543.

**Table 1:** Overview of treatment-emergent adverse events occurring in ≥ 10% of patients.

	MDS (*N* = 33) CTCAE Grade			AML (*N* = 7) CTCAE Grade			Total (*N* = 40) CTCAE Grade		
Adverse Event	1–2 n	3–5 n	Any n (%)	1–2 n	3–5 n	Any n (%)	1–2 n	3–5 n	Any *n* (%)
*Number (%) of Subjects With Any TEAE*	**4**	**29**	**33 (100)**	**2**	**5**	**7 (100)**	**6**	**34**	**40 (100)**
Anemia	0	18	18 (54.5)	0	2	2 (28.6)	0	20	20 (50.0)
Thrombocytopenia	1	10	11 (33.3)	0	1	1 (14.3)	1	11	12 (30.0)
Nausea	9	0	9 (27.3)	2	0	2 (28.6)	11	0	11 (27.5)
Diarrhea	7	1	8 (24.2)	2	1	3 (42.9)	9	2	11 (27.5)
Fatigue	6	0	6 (18.2)	2	0	2 (28.6)	8	0	8 (20.0)
Constipation	6	1	7 (21.2)	0	0	0	6	1	7 (17.5)
Decreased appetite	6	0	6 (18.2)	1	0	1 (14.3)	7	0	7 (17.5)
Dizziness	7	0	7 (21.2)	0	0	0	7	0	7 (17.5)
Dyspnea	4	0	4 (12.1)	3	0	3 (42.9)	7	0	7 (17.5)
Pneumonia	2	3	5 (15.2)	1	1	2 (28.6)	3	4	7 (17.5)
Headache	2	0	2 (6.1)	4	0	4 (57.1)	6	0	6 (15.0)
Contusion	5	0	5 (15.2)	1	0	1 (14.3)	6	0	6 (15.0)
Neutropenia	0	5	5 (15.2)	0	1	1 (14.3)	0	6	6 (15.0)
Cough	4	0	4 (12.1)	2	0	2 (28.6)	6	0	6 (15.0)
Oedema peripheral	5	0	5 (15.2)	1	0	1 (14.3)	6	0	6 (15.0)
Pyrexia	5	0	5 (15.2)	1	0	1 (14.3)	6	0	6 (15.0)
Epistaxis	4	0	4 (12.1)	1	0	1 (14.3)	5	0	5 (12.5)
Hypertension	2	2	4 (12.1)	1	0	1 (14.3)	3	2	5 (12.5)
Back pain	4	0	4 (12.1)	0	0	0	4	0	4 (10.0)
Hypophosphatemia	3	0	3 (9.1)	1	0	1 (14.3)	4	0	4 (10.0)
Petechiae	3	0	3 (9.1)	1	0	1 (14.3)	4	0	4 (10.0)

*AML* acute myeloid leukemia, *CTC* common terminology criteria of adverse events, *MDS* myelodysplastic neoplasm, *TEAE* treatment-emergent adverse events.

The median duration of treatment with PRT543 was 3.6 months (R: 0.4–17.6 months). The most common reasons for treatment discontinuation were progressive disease (*n* = 22 patients; 55.0%), AEs (*n* = 6; 15.0%), and lack of efficacy (*n* = 4; 10.0%).

Four patients were unevaluable for response due to absence of a repeat BM biopsy and were considered non-responders. Best response by central assessment was 1 HI-erythroid (HI-E) among 18 patients with lower-risk MDS and mCR in 1 patient and HI in 2 patients (1 HI-E and 1 trilineage HI) among 15 patients with higher-risk MDS and MDS/MPN overlap. One out of 7 AML patients achieved CRi and 1 additional patient with SD achieved platelet transfusion independence from a baseline of requiring 9 units of platelets over 8 weeks preceding the trial. All other patients had SD or progressive disease as best response. Supplementary Table 7 provides additional disease characteristics of the responding patients. On serial molecular testing we found no change in mean VAF of splicing factor mutations (Supplementary Fig. 4). Individual patient outcomes are shown in Fig. 1A.

**Fig. 1:**
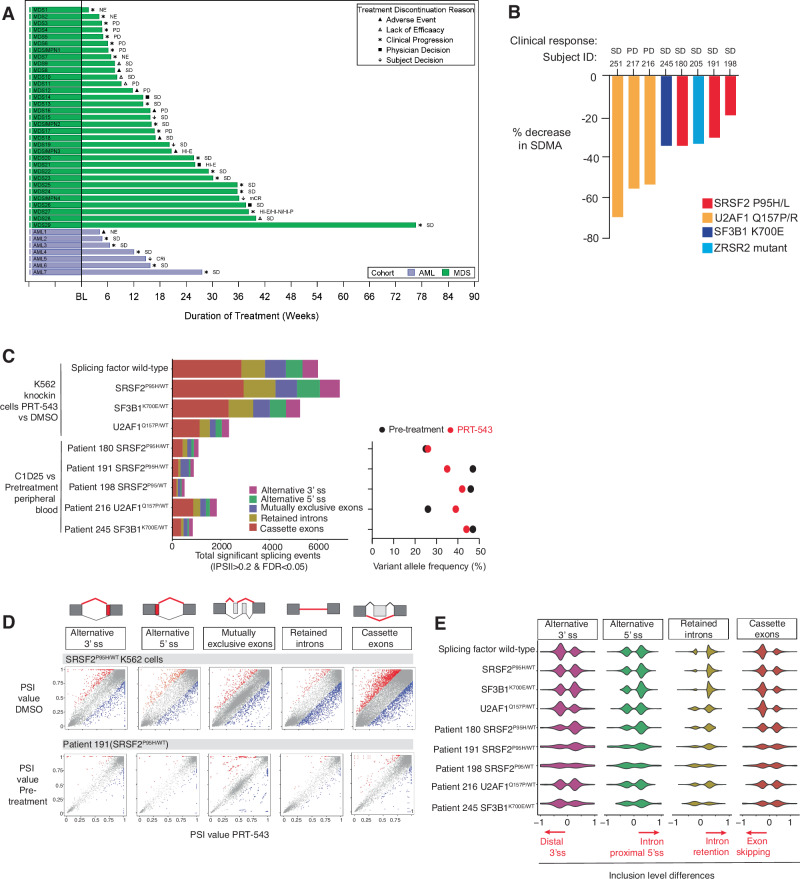
Swimmer’s plot of patients treated with PRT543 and In vitro and in vivo splicing changes induced by PRT543. **A** Duration of study treatment in weeks of individual patients treated with PRT543 is shown. Best response and reason for treatment discontinuation are also shown. Abbreviations: AML acute myeloid leukemia, CRi complete remission with incomplete hematologic recovery, HI-E hematologic improvement – erythroid, HI-N hematologic improvement – neutrophil, HI-P hematologic improvement – platelets, mCR marrow complete remission, MDS myelodysplastic neoplasms, NE not evaluable, PD progressive disease, SD stable disease. **B** Serum levels of symmetric dimethylarginine (SDMA) decrease post-treatment (C1D25) vs. pre-treatment levels in 8 patients treated with PRT543. Colors denote splicing factor mutation type. **C** Enumeration of statistically significant alternative splicing events (defined as absolute value of percent spliced-in (PSI) > 0.2 and FDR < 0.05) on PRT543 treatment versus DMSO in cell lines or pre-treatment in patient samples. Colors denote distinct categories of RNA splicing events. Right, changes in variant allele fractions of splicing factor mutations in patient samples pre-treatment (black) vs. on PRT543 (red). **D** Scatter plots of differential alternative splicing events in the cell line with *SRSF2*^*P95H*^ knock-in mutation (top*)* and peripheral blood mononuclear cells of a patient with a *SRSF2*^*P95H*^ mutation (bottom), where y-axis indicates PSI in the control condition (DMSO for cell line and pre-treatment for patient sample) and x-axis indicates PSI on treatment with PRT543. The legend at the top indicates alternative splicing in red and constitutive splicing in gray for the different categories of splicing events. **E** Violin plots of the distribution of exon inclusion level differences for different categories of splicing events in cell lines and patient samples (C1D25) on treatment with PRT543 compared to DMSO or pre-treatment.

In 8 paired pre- and post-treatment samples we found a mean reduction in serum SDMA by 41.9%, which confirmed PRMT5 inhibition in patients (Fig. 1B). We next evaluated the impact of PRT543 treatment on splicing in PB MNCs prior to therapy and on treatment at C1D25 (Supplementary Table 8 lists patient and disease characteristics). In parallel, we treated leukemia cell lines with the same mutations in *SF3B1*, *SRSF2*, and *U2AF1* as seen in patients with DMSO as control or PRT543 at doses (2–10 μM over 24 h) identified as having anti-cancer effect in prior studies.

PRT543 induced extensive global changes in splicing regardless of splicing mutational status, with the greatest number of significant events in the cell line with *SRSF2*^P95H^ (Fig. 1C). The number of statistically significant RNA splicing events induced by PRT543, was on average 80% less than those seen in cell lines in response to PRT543. Intron retention and exon skipping represented the most prominent alternative splicing events across patient samples (Fig. 1D, E). There were several intron retention events in cell lines also observed in patients, such as in *MIB2*, which encodes an E3 ubiquitin protein ligase, and *SNHG12*, which encodes a long non-coding RNA (Supplementary Fig. 5A). There was modest overlap in significant alternative splicing events across cell lines with WT and different splicing factor mutations (Supplementary Fig. 5B). Recurrent splicing events in all cell lines affected genes involved in interferon-α response, MYC targets, and DNA repair (Supplementary Fig. 5C). Based on gene expression, cell lines and patient samples clustered distinctly from one another regardless of genotype or drug treatment (Supplementary Fig. 5D, E). Gene set enrichment analysis of all differentially expressed genes post- vs. pre-treatment in patient samples revealed upregulation of KRAS signaling, MYC targets, and inflammatory response but did not reach statistical significance (Supplementary Fig. 5F).

In this dose-escalation/expansion trial of PRT543 in patients with R/R myeloid malignancies we demonstrated the safety of PRT543 monotherapy and observed clinical efficacy especially among patients with *SRSF2* mutations with HI and CRi in a subset of heavily pretreated patients. The results indicate an effect of PRT543 specific to the leukemic cell clone leading to the restoration of normal hematopoiesis.

We also present the crystal structure and functional characterization of PRT543 and demonstrate that it exerts its anti-tumor effect as a SAM and MTA-competitive and substrate-uncompetitive inhibitor. We identify for the first time in patients that treatment with PRT543 leads to target engagement and global disruption in splicing. However, the extent of splicing perturbations in patient samples was substantially less than in cell lines, which might be an explanation for the limited clinical efficacy. Pharmacodynamics could also explain differences in abundance of splicing changes induced by PRT543 in cell lines versus patient samples. We used a dose of 10 μM PRT543 in cell lines, which was higher than peak concentrations of PRT543 reached in patients (1.48 µM) [9]. While efficacy of PRT543 monotherapy was modest, newer generation MTA-cooperative PRMT5 inhibitors are now in phase I trials in patients with solid tumors with MTAP deletions [14]. Additionally, it will be helpful for future studies to evaluate rational combinatorial therapies with PRMT5 inhibitors to enhance clinical benefit from this agent.

## Supplementary information

Supplemental materials

Supplemental Figure 1. PRT543 is a potent and selective PRMT5 inhibitor.

Supplemental Figure 2. Anti-tumor activity of PRT543 in vivo.

Supplemental Figure 3. Baseline cytogenetic and molecular characteristics and association with response.

Supplemental Figure 4. Maximum change in variant allele fraction (VAF) of splicing mutations.

Supplemental Figure 5. Aberrant splicing events induced by PRT543.

## Data Availability

Deidentified original data can be requested from the corresponding author.
