# Supplementary material for: Phase Ib study of PRT543, an oral protein arginine methyltransferase 5 (PRMT5) inhibitor, in patients with advanced splicing factor-mutant myeloid malignancies
Source: Leukemia. 2025 Jan 24;39(3):765–9. doi: 10.1038/s41375-025-02515-8 (PMC11879867; doi:10.1038/s41375-025-02515-8)
Supplement: Supplementary file 1 — Supplemental materials [file 41375_2025_2515_MOESM1_ESM.docx]

- **Supplement -**

**Phase Ib study of PRT543, an oral protein arginine methyltransferase 5 (PRMT5) inhibitor, in patients with relapsed or refractory, splicing factor-mutant myeloid malignancies**

Jan Philipp Bewersdorf ^1,2,*^, Xiaoli Mi^1,*^, Bin Lu^1^, Andrew Kuykendall^3^, David Sallman^3^, Manish Patel^4^, Don Stevens^5^, Alexander Philipovskiy^6^, Grerk Sutamtewagul^7^, Lucia Masarova^8^, Gina Keiffer^9^, Amit Verma^10^, Neha Bhagwat^11^, Min Wang^11^, Andrew Moore^11^, Joseph Rager^11^, Diane Heiser^11^, Sunhee Ro^11^, Wan-Jen Hong^11^, Omar Abdel-Wahab^1,12,#^, and Eytan M. Stein^1,^

**Affiliations:**

1: Department of Medicine; Leukemia Service, Memorial Sloan Kettering Cancer Center, New York, NY

2: Yale University and Yale Cancer Center, New Haven, CT

3: Moffitt Cancer Center, Tampa, FL, USA;

4: Florida Cancer Specialists, Sarasota, FL, USA;

5: Norton Cancer Institute, Louisville, KY, USA;

6: Florida Cancer Specialists, Lake Mary, FL, USA;

7: University of Iowa, Iowa City, IA, USA;

8: MD Anderson Cancer Center, Houston, TX;

9: Department of Medical Oncology, Sidney Kimmel Cancer Center, Thomas Jefferson University Hospital, Philadelphia, PA, USA;

10: Montefiore Einstein Comprehensive Cancer, Bronx, NY, USA;

11: Prelude Therapeutics, Wilmington, DE, USA

12: Molecular Pharmacology Program, Sloan Kettering Institute, Memorial Sloan Kettering Cancer Center, New York, NY

*****These authors contributed equally and serve as co-first authors

# These authors contributed equally and serve as co-senior authors

**Corresponding authors:**

Omar Abdel-Wahab

Email: [abdelwao@mskcc.org](mailto:abdelwao@mskcc.org)

Phone: 347-821-1769

Eytan M. Stein

Email: [steine@mskcc.org](mailto:steine@mskcc.org)

Phone: 646-608-3749

**Supplemental Methods:**

*Patients:*

This was a multicenter, open-label, sequential cohort, dose-escalation/expansion phase I study of PRT543 monotherapy in patients with advanced solid tumors, non-Hodgkin lymphoma (NHL), and selected myeloid malignancies. Only data from patients with AML, MDS, and MDS/MPN overlap are reported here. Results for the other cohorts will be reported separately. Preliminary data have been presented previously.^12, 13^ All patients provided written informed consent prior to enrolling in the study. The trial protocol was approved by the institutional review board at the participating sites and was conducted in accordance with the Good Clinical Practice guidelines of the International Council for Harmonization and the principles of the Declaration of Helsinki.

Adult patients (≥18 years) were included. Patients in the dose escalation phase had to have relapsed or refractory (R/R) MDS, MPN, or MDS/MPN overlap but were not required to have splicing factor mutations. Based on promising efficacy in patients with splicing factor-mutant disease, patients were required to have ≥1 mutation in an RNA splicing factor gene for enrollment in the dose expansion cohort. A full list or inclusion and exclusion criteria is provided in the **Supplemental Table 1.**

*Study Design and Interventions:*

Dose escalation occurred using an accelerated dose escalation design initially followed by a conventional 3+3 design until the recommended phase 2 dose (RP2D) was identified. In the absence of grade 2 or higher toxicities, doses were escalated in increments of 100% in single patient cohorts. When any single patient experienced a grade 2 or higher toxicity, the trial reverted to a standard 3+3 design, starting at the dose where the single patient experienced the toxicity.

The following dose levels were tested among patients with myeloid malignancies: (I) PRT543 monotherapy (dosing 5 – 40mg) on day 1 and 4 of each week of the 28-day cycle; (II) 40mg PRT543 monotherapy on days 1, 3, and 5 of each week; (III) 20mg PRT543 monotherapy once daily continuously over the course of the 28-day cycle and (IV) 35mg PRT543 monotherapy on days 1, 2, 3, 4, and 5 of each week.

Dose expansion was performed in multiple parallel cohorts at the RP2D identified during the dose escalation phase. Patients with lower-risk, splicing factor mutant MDS enrolled in the dose-expansion phase received treatment at the lowest cumulative weekly dose identified during the dose-escalation phase with escalation to the highest cumulative weekly dose after 2 cycles. Patients with high-risk MDS, MDS/MPN overlap, and AML started treatment at the highest cumulative weekly dose identified as safe during the dose escalation phase.

*Definition of dose-limiting toxicities (DLT)*

Non-hematologic dose-limiting toxicities (DLT) were defined as grade 3 or higher AST or ALT elevations or evidence of hepatotoxicity according to Hy's Law, grade 4 vomiting or diarrhea, grade 3 nausea, vomiting, or diarrhea that does not resolve or reduce to grade 1 within 48 hours of medical management, and any other grade 3 or higher non-hematologic toxicity. As patients with advanced myeloid malignancies frequently have significant hematologic abnormalities at baseline, DLT was defined as any of the following: grade 4 neutropenia lasting ≥7 days, febrile neutropenia (fever of ≥38.5°C in the setting of grade 3 or 4 neutropenia), grade 4 anemia not explained by underlying disease, grade 4 thrombocytopenia (platelet count of <25,000/µL for patients with baseline platelets >50,000/µL) of any duration or grade 3 thrombocytopenia (platelet count of <50,000/µL) with clinically significant bleeding or requiring platelet transfusion. Isolated grade 3 or 4 lab abnormalities (single day abnormalities) were not considered DLTs.

*Patient mutational analysis*

Mutational analysis of pre- and post-PRT543 peripheral blood mononuclear cells (PB MNCs) or bone marrow mononuclear cells (BM MNCs) from patients was performed using the Qiagen QIASeq Human Comprehensive Cancer (275 gene) targeted next-generation sequencing platform.

*Measurement of serum symmetric dimethylarginine levels:*

Serum concentrations of SDMA in patients treated with PRT543 were measured using a liquid chromatography-tandem mass spectrometry (LC-MS/MS) assay. Briefly, samples (50 µL) were prepared for analysis by precipitation with a 1:1 (v:v) mixture of methanol and acetonitrile containing 0.1% formic acid and the internal standard, SDMA-d_6_ (10 ng/mL).  Following centrifugation, the resulting supernatants were directly injected into an LC-MS/MS.  Chromatography was conducted on a Shimadzu HPLC equipped with a Waters XBridge Amide, 50 x 2.1 mm i.d., 3.5 µm column in hydrophilic interaction liquid chromatography (HILIC) mode utilizing a gradient of 95:5 (v:v) acetonitrile:200 mM ammonium formate, pH 3 and 50:45:5 (v:v:v) acetonitrile:water:200 mM ammonium formate, pH 3.  Detection was conducted on an ABSciex API4000 triple mass spectrometer in the positive ion mode.  Mass transitions of 203.2 > 172.2, and 209.2 > 175.2 are monitored for SDMA and SDMA-d6, respectively.  Following a 5 µL injection, linear calibration ranges of 1 to 250 ng/mL were achieved for SDMA.

*RNA-sequencing library preparation and sequencing:*

For patient PBMNC RNA sequencing (RNA-seq), RNA was extracted using the Qiagen RNeasy extraction kit, according to the manufacturer’s instructions. For cell lines, RNA was extracted using similar methods from either K562 cells wild-type for splicing factor mutations or with knockin of a single allele mutation that encodes SF3B1^K700E^, SRSF2^P95H^, or U2AF1^Q157P^ after 24 hours of DMSO or PRT543 (2μM was used for SF3B1^K700E^ and U2AF1^Q157P^ knockin cells while 10uM was used for wild-type and SRSF2^P95H^ mutant cells) treatment. A minimum of 500ng of high-quality RNA (as determined by Agilent Bioanalyzer) per sample was used as input for library preparation. Poly(A)-selected, strand-specific (dUTP method) Illumina libraries were prepared by the Integrated Genomics Operation (IGO) at Memorial Sloan Kettering with a modified TruSeq protocol and sequenced on the Illumina HiSeq 2000 to obtain ∼100M 2x101 bp paired-end reads per sample.

*RNA sequencing analysis:*

Prior to mapping, raw FASTQ files were trimmed using Trim_galore (v0.6.7) to remove residual Illumina adapter and/or low quality (Q<15) sequences. Trimmed sequencing reads were then aligned to the human hg38 reference genome (GENCODE, release 45, GRCh38.p14) using STAR (v2.7.3a). Samtools (v1.9) was used to convert SAM files to BAM files, as well as sorting and indexing.

Differential alternative splicing events were detected using Multivariate Analysis of Transcript Splicing (rMATS, v4.1.1) using the GENCODE (release 45) GTF annotation for GRCh38. rMATS was run with the –novelSS option to also identify novel, unannotated splice junctions not represented within the GTF annotation.^1-3^ Enumeration of isoform counts was performed using only reads that span the splice junction directly. Pairwise sample comparisons were performed and significant differential alternative splicing events were defined as those with greater than an absolute difference of 20% in isoform expression (|PSI|>0.2) and a false discovery rate of less than 5% (FDR<0.05). To identify robust AS events in all pairwise comparisons, junctions were first pre-filtered to remove those that possessed fewer than 30 reads on average. Shared and distinct splicing changes on PRT543 compared to DMSO were identified in cell lines with wild-type and splicing factor mutations. The functions of recurrently alternatively spliced genes were analyzed using EnrichR (v3.2) with MSigDB Hallmark (2020) as the gene-set library.^4-6^ All analyses were conducted within the R Programming environment with tools from Bioconductor.^7^ The visualizations were created using the dplyr, tidyverse, ggplot2, ggvenn, and ComplexHeatmap packages.

For gene expression, read counts across genomic features (exons) were generated using featureCounts (part of the subread package; v2.0.4) with the following parameters: -p -T 8 -F GTF -t exon.^8^ The resultant counts matrix was used as input for differential gene expression analysis, which was performed using DESeq2 (v1.30.1).^9,10^ Data visualization and figure generation was performed in Rstudio (v1.3.1073) using the following packages: ggplot2 (v3.3.5) and complexHeatmap (v2.6.2). The functions of differentially expressed genes among all paired post-treatment vs. pre-treatment patient samples were analyzed using Fast Gene Set Enrichment Analysis (v.1.21.2) with MSigDB Hallmark (2023) as the gene-set library.

*PRMT5:MEP50 purification and crystallization*

The human PRMT5 construct was designed with a FLAG fusion in the N-terminal. The human MEP50 construct was designed with an 8*His fusion in the N-terminal. The coding sequences of hPRMT5 and hMEP50 were cloned into a pFastbac vector. Standard baculovirus expression using a modified version of the Bac-to-Bac system protocol (LifeTechnologies) was used to generate virus for each clone. For protein expression, SF9 cells (3.5x10^6^ cells/mL) were infected with hPRMT5 and hMEP50 viruses, cultured at 27˚C. Cells were harvested by centrifugation at 4°C, 900xg, and stored at -80˚C until purification. Harvested cells were resuspended in lysis buffer (50 mM Tris pH 8.0, 250 mM NaCl, 1 mM TECP, 0.3mM PMSF, 1 table of complete protease cocktail inhibitor (Roche)/50ml lysate, 1μL Benzonase/100mL) and sonication. The homogenate was clarified by centrifugation for 60min at 40,000 g. The supernatant was gently shaken with Ni-NTA resin for 3h. The hPRMT5/hMEP50 complex was eluted with 50 mM Tris pH 8.0, 250 mM NaCl, 1 mM TECP, 0.3mM PMSF, 250mM imidazole, followed purified by FLAG resin and SEC chromatography (Superdex200 increase, Cytiva), Fractions containing PRMT5:MEP50 were concentrated to 10-20 mg/ml for crystallization.

The hPRMT5:hMEP50 complex was incubated with 2mM PRT543 at 4°C overnight. Co-crystals were grown at a concentration of 20 mg/ml (10 mM HEPES, 150 mM NaCl, 1 mM TCEP, 10% Glycerol pH8.0) by hanging-drop vapor diffusion. Crystals were cryo-protected in the reservoir solution supplemented with 20% (vol/vol) glycerol and flash-frozen in liquid nitrogen for data collection.

*Structure Determination*

| PDB ID | 20240307_VIVA214-10 |
| --- | --- |
| Data collection | |
| Space Group | I222 |
| Cell dimensions |  |
| a, b, c (Å)  α, β, γ (°) | 102.567 139.202 178.548  90.00 90.00 90.00 |
| Resolution(Å) | 54.89– 2.50 (2.59 – 2.50)**^a^** |
| Total observations | 619876 (66944) |
| Unique reflection | 44312 (4599) |
| R_merge_ (%)^b^ | 5.8 (58.0) |
| I/σI | 25.4 (2.3) |
| Completeness (%) | 99.6 (99.4) |
| Redundancy | 14.0(14.6) |
| Refinement | |
| Resolution (Å) | 54.95– 2.50 |
| No. reflections | 42104 |
| R_work_^c^/R_free_^d^ | 0.205/0.259 |
| No. atoms |  |
| Protein | 7421 |
| Ligand/ion | 44 |
| Water | 266 |
| B-factors | |
| Protein | 79.47 |
| Ligand/ion | 58.87 |
| Water | 62.5 |
| R.m.s. deviations^e^ |  |
| Bond lengths (Å) | 0.0028 |
| Bond angles (°) | 1.2509 |
| Ramachandran plot^f^ |  |
| Most favored (%) | 95.29 |
| Outlier (%) | 0.21 |

Complete diffraction datasets for the best crystals were collected at beam line Diamond Light Source at the UK’s national synchrotron science facility, at 100 K with wavelength 0.97627. Datasets were processed using the program XDS1. Five percent of the data were randomly selected for the R-free calculation. The initial structure solution of PRMT5 and MEP50 complex in the I222 space group was solved by molecular replacement using 7mx7 from the RCSB database as the search model. Phaser4 was used for model building. The model was refined to 2.5 Å using Refmac5 from CCP4 program suite5 and rebuilt with Coot6. The quality of the models was checked using MolProbity7.

*PRMT5/MEP50 enzymatic assay*

Assay buffer containing the PRMT5/MEP50 complex (2.5 nM), 3H-labeled SAM (1 µM) and PRT543 was incubated for 20 min at 37°C. The reaction was then initiated by addition of 300 nM histone H4 biotinylated peptide substrate (SGRGKGGKGLGKGGAKR HRKVLRGSGSK-Biotin). The reaction was stopped after 30 min incubation at 37°C with the addition of 8M guanidine HCl. Streptavidin YSI SPA beads were added, and the plate was incubated for 1 hour in a Microbeta2 (PerkinElmer) plate chamber before reading.

*Jump-dilution catalytic assay*

The PRMT5/MEP50 complex was pre-incubated with PRT543 or without any inhibitor for 30 min at 37°C to allow the formation of the enzyme-inhibitor complex (EI complex). The EI complex was then dialyzed for 1 hour at room temperature to remove extra inhibitor, and diluted in reaction buffer containing 1 µM 3H-labeled SAM, and 300 nM of histone H4 biotinylated peptide substrate and the reaction was run for 1 hour at 37°C. During the reaction process, a small aliquot of the reaction mixture was taken out and quenched with 8M guanidine HCl at specified time points. Streptavidin YSI SPA beads were added, and the plate incubated in a Microbeta2 (PerkinElmer) plate chamber for 1 hour before reading.

*Mode of binding assay*

The inhibition modality of PRT543 was investigated by plotting the IC_50_ values against the concentration of SAM (expressed as [SAM]/K_M, SAM_), and Histone H4 peptide (expressed as [substrate]/ K_M, peptide_), respectively. The IC_50_ values were determined as described in the enzymatic assay protocol and plotted using the following equations.

$${IC}_{50}=K_{i}\left( 1+\frac{\left[ S \right]}{K_{M}} \right)$$

$${IC}_{50}={\alpha K}_{i}(1+\frac{K_{M}}{\left[ S \right]})$$

Where:

[S]=substrate peptide or SAM concentration

*K*_M_ = Michaelis constant

*K*_i_  = inhibition constant for binding to free enzyme

α *K*_i_ = inhibition constant for binding to enzyme-substrate complex.

*PRT543 selectivity screen*

PRT543 activity was assessed by a radiometric assay (HotSpot) in which 37 methyltransferases with their corresponding substrates were preincubated with 10 µM PRT543 for 20 minutes at room temperature before addition of H3-labelled SAM to initiate the reaction. After 1 hour incubation at 30 ⁰C, the reaction mixture was delivered to filter-paper for detection. The selectivity was represented by % activity at the testing concentration, where 100% indicates no inhibition and 0% indicates 100% inhibition.

*Western blotting*

Cells were lysed in 4% SDS and probed with sDMA and β-Actin antibodies (sDMA: Cell signaling, Catalog #: 13222, 1:3,000; β-Actin: sigma, Catalog #:A2228, 1:5,000). Chemiluminescent signals were captured using FluorChem HD2 imaging system (ProteinSimple), and the sDMA (SmD3me2s at ~ 15 kDa) as well as β-Actin bands were quantified by ImageJ and analyzed.

*Xenograft studies and OncoPanel™ cell proliferation assay*

1 × 10^7^ tumor cells (0.1 mL cell suspension) were injected subcutaneously in the right flank of 8-week-old female SCID Beige (SET2) or CB.17 SCID (HEL92.1.17) mice. Drug treatment was initiated when tumor volume was 100 - 150 mm^3^. PRT543 or vehicle (0.5% carboxymethyl cellulose (CMC), 0.5% Tween-80 in sterile water) was administered daily by oral gavage. Cells were incubated with PRT543 for 10 days, with media and compound replacement done on Day 7. At end of treatment, cells were fixed and stained to allow fluorescence imaging of nuclei. Automated fluorescence microscopy was carried out using a Molecular Devices ImageXpress Micro XL high-content imager, and images were collected with a 4X objective. 16-bit TIFF images were acquired and analyzed with MetaXpress 5.1.0.41 software.

*Sample size:*

No power calculation was required for the dose-escalation phase and patients were enrolled in a 3+3 design followed by a dose confirmation until the RP2D was achieved. For the expansion part of the study, each cohort enrolled up to 20 efficacy-evaluable patients. The sample size of 20 efficacy-evaluable patients for each of the expansion cohorts was determined based on a one-sided exact test with a significance level (alpha) of 0.10 (one-sided). The null hypothesis that the response rate is 0.05 was tested against a one-sided alternative of 0.19 response rate. The null hypothesis would be rejected if 3 or more responders out of the 20 patients were observed. This test yielded an 80% power under the alternative hypothesis.

*Statistical analysis:*

All patients who received any study drug were evaluated for safety and efficacy. Patients who received any study drug and were not evaluable for response were considered as non-responders for the efficacy analysis. Continuous variables, including baseline characteristics, were summarized as mean, standard deviation, median, and range. Categorical/discrete variables were summarized using frequency tables. The calculation of adverse event (AE) incidence was based on the number of patients per AE category. Patient with multiple AEs classified to the same category were tabulated under the worst toxicity grade for that AE category.

Frequency counts, percentages, and 95% confidence intervals (95% CI) were used to define the response rate. Time-to­event data were analyzed using the Kaplan-Meier method and were summarized as median time to event and 95% CI. PFS was calculated for all patients from the first administration of study drug until death or until disease progression. Interim analyses for toxicity with predefined stopping criteria were performed after enrollment of 5, 10, 15, and 20 patients in each expansion cohort. There was no interim analysis for efficacy.

**Supplemental Table 1. Overview of key inclusion and exclusion criteria.**

| Inclusion Criteria | Exclusion Criteria |
| --- | --- |
| ≥ 18 years of age | Uncontrolled CNS disease. |
| Patients must be ambulatory with an Eastern Cooperative Oncology Group (ECOG) Performance Score of 0 or 1. | Patients who require pharmacologic doses of glucocorticoids (> I 0mg of prednisone or equivalent) require Sponsor consultation and approval prior to enrollment. |
| Adequate hepatic function as evidenced by AST and ALT levels ≤ 2.5 x ULN for the reference lab AND serum bilirubin levels ≤ 1.5 x ULN for the reference lab OR a direct bilirubin ≤ ULN for subjects with total bilirubin levels > 1.5 x ULN (exceptions can be made for subjects with Gilbert's syndrome following consultation with Sponsor). | Patients with uncontrolled inflammatory disorders of the gastrointestinal tract, or patients with uncontrolled GI malabsorption. |
| Must have adequate renal function as evidenced by serum creatinine values ≤ 1.5 x ULN for the reference lab or a calculated creatinine clearance of ≥ 60 mL/min according to the Cockroft-Gault equation. | Mean QTcF interval of> 470 msec following 3 ECGs conducted 5 minutes apart, unless NCS per Investigator. |
| On prior investigational agents must wait at least 5 half-lives of the agent in question, or 28 days, whichever is shorter before enrollment into the trial. A shorter wash-out period may be considered for prior therapy with a short half­life (e.g. <5 hours) pending SPONSOR approval. | Any clinically significant graft-versus-host disease (GVHD) secondary to prior allogenic transplant. Patients must be > 90 days from transplant.  NOTE: Topical corticosteroids for minor skin rash is acceptable. Prior solid organ transplant is acceptable provided the patient is on no immunosuppressive therapy. Should the patient be on immunosuppressive therapy for GVHD, Sponsor consultation is required. |
| Must be recovered from the effects of any prior systemic therapy, radiotherapy or surgery (i.e., toxicity no worse than Grade 1 ). | Patients who require treatment with strong inhibitors or inducers of CYP3A4 for which there are no therapeutic substitutions |
| Female patients of childbearing potential must have a negative pregnancy test (urine/serum) within 7 days of the start of treatment. | Patients who require treatment with MAO inhibitors. |
| Splicing factor-mutant, lower-risk MDS cohort:  a. Disease should be either International Prognostic Scoring System-Revised [IPSS-R] very low risk, low risk or intermediate risk.  b. ≥1 Splicing Mutation (e.g. U2AF1, SRSF2, ZRSR2, SF3B1, EIF1AX).  c. Must have been receiving regular red-cell transfusions defined as ≥2 RBC U/8 weeks during the 16 weeks prior to study entry.  d. Must have had either an inadequate response to prior treatment with an erythropoiesis-stimulating agent (ESA), be intolerant of ESAs or have serum erythropoietin >200 U/L.  e. Prior treatment with a hypomethylating agent is acceptable.  f. Prior treatment with luspatercept is acceptable providing there is a 28-day washout prior to starting study therapy.  g. Patients with del(5q) must have been previously treated with lenolidomide. |  |
| Splicing Mutant Intermediate or High Risk MDS, CMML, or AML Cohort:  a. ≥1 Splicing Mutation (e.g., U2AF1, SRSF2, ZRSR2, SF3B1, EIF1AX).  b. Patients with higher risk MDS, defined as intermediate, high, or very high risk by International Prognostic Scoring System-Revised [IPSS-R] criteria must have failed prior therapy with a hypomethylating agent administered either as monotherapy or in combination.  c. Patients with higher risk CMML, defined as intermediate-2 or high risk per CMML-specific prognostic scoring system (CPSS) or clinical/molecular CPSS (CPSS-mol) criteria, must have failed prior therapy with a hypomethylating agent.  e. Patients with AML:   - Must have relapsed or refractory AML. Refractory AML is defined as (a) for patients who received induction chemotherapy, having persistent AML after ≥ 2 cycles of intensive induction or (b) for patients unfit for induction chemotherapy, having relapsed or been refractory to treatment with a single agent hypomethylating agent or low dose cytarabine (at least two cycles) or a an HMA/LDAC in combination with venetoclax (at least one cycle) or another standard of care therapy ( e.g. gemtuzumab ozogamicin, glasdegib/LDAC). - Patients with targetable mutations [IDHl and 2, FLT3 (ITD, and TK.D)] must have failed appropriate therapy prior to enrollment. |  |

**Supplemental Table 2. Selectivity of PRT543 against a panel of 37 methyltransferases.**

| **Methyltransferases** | **% Activity at 10uM of PRT543** |
| --- | --- |
| ASH1L | 89.945 |
| DNMT1 | 99.27 |
| DNMT3a | 86.785 |
| DNMT3b | 99.96 |
| DNMT3b/3L | 104.12 |
| DOT1L | 83.48 |
| EZH1 Complex | 93.18 |
| EZH2 Complex | 103.17 |
| G9a | 92.06 |
| GLP | 101.335 |
| METTL21A-GST | 98.625 |
| MLL1 Complex | 83.15 |
| MLL2 Complex | 98.67 |
| MLL3 Complex | 101.04 |
| MLL4 Complex | 82.355 |
| NRMT1 | 88.825 |
| NRMT2 | 89.36 |
| NSD1 | 98.97 |
| NSD2 | 89.81 |
| NSD3 | 92.07 |
| PRDM9 | 80.635 |
| PRMT1 | 96.635 |
| PRMT3 | 82.77 |
| PRMT4 | 63.495 |
| PRMT5/MEP50 Complex | -0.14 |
| PRMT6 | 95.185 |
| PRMT7 | 91.175 |
| PRMT8 | 91.56 |
| SET1b Complex | 105.505 |
| SET7/9 | 90.24 |
| SET8 | 78.73 |
| SETD2 | 107.795 |
| SMYD1 | 90.31 |
| SMYD2 | 93.6 |
| SUV39H1 | 90.46 |
| SUV39H2 | 96.535 |
| SUV420H1TV2 | 93.165 |

**Supplemental Table 3: Cell lines tested in *OncoPanel™* cell proliferation assay**

| **Cell Line** | **IC50 (nM)** | **Tissue type** |
| --- | --- | --- |
| T24 | 80.8 | Bladder |
| SCaBER | 112 | Bladder |
| BFTC-905 | 117 | Bladder |
| HT1376 | 122 | Bladder |
| TCCSUP | 123 | Bladder |
| J82 | 161 | Bladder |
| 5637 | 264 | Bladder |
| HT-1197 | 309 | Bladder |
| MDAMB468 | 54.5 | Breast |
| MDAMB231 | 137 | Breast |
| MDAMB436 | 153 | Breast |
| Hs578T | 314 | Breast |
| BT549 | 366 | Breast |
| BT20 | 448 | Breast |
| A172 | 36 | Brain |
| DBTRG-05MG | 60.2 | Brain |
| T98G | 71.1 | Brain |
| U-87 MG | 155 | Brain |
| DK-MG | 196 | Brain |
| U-118 MG | 199 | Brain |
| CCF-STTG1 | 244 | Brain |
| U-138MG | 253 | Brain |
| M059J | 280 | Brain |
| SW1783 | 353 | Brain |
| OE33 | 66 | Head & Neck |
| Detroit562 | 95.1 | Head & Neck |
| OE21 | 108 | Head & Neck |
| A253 | 130 | Head & Neck |
| Cal27 | 135 | Head & Neck |
| FaDu | 143 | Head & Neck |
| OE19 | 182 | Head & Neck |
| CRO-AP2 | 34.2 | Blood |
| MV-4-11 | 42.8 | Blood |
| SR | 48 | Blood |
| HT | 68.7 | Blood |
| TUR | 70.7 | Blood |
| Hs 611.T | 87.6 | Blood |
| ST486 | 90.2 | Blood |
| DOHH-2 | 99.9 | Blood |
| MC116 | 108 | Blood |
| MX1 | 120 | Blood |
| Ramos (RA 1) | 130 | Blood |
| Daudi | 138 | Blood |
| NU-DUL-1 | 141 | Blood |
| Raji | 143 | Blood |
| Jiyoye | 150 | Blood |
| SU-DHL-10 | 156 | Blood |
| SU-DHL-8 | 177 | Blood |
| NAMALWA | 181 | Blood |
| BC-1 | 191 | Blood |
| SU-DHL-4 | 192 | Blood |
| DB | 196 | Blood |
| Thp1 | 196 | Blood |
| EB-3 | 206 | Blood |
| RPMI 6666 | 255 | Blood |
| EB2 | 259 | Blood |
| JeKo-1 | 703 | Blood |
| U-937 | 10000 | Blood |
| SNU423 | 101 | Liver |
| HuCCT1 | 147 | Liver |
| HLF | 264 | Liver |
| OCUG1 | 289 | Liver |
| HUH6Clone5 | 290 | Liver |
| HepG2 | 291 | Liver |
| HLE | 10000 | Liver |
| DMS114 | 100 | Lung |
| A549 | 132 | Lung |
| NCI-H520 | 144 | Lung |
| NCI-H596 | 213 | Lung |
| SW900 | 253 | Lung |
| NCI-H69 | 271 | Lung |
| NCIH446 | 474 | Lung |
| SHP-77 | 615 | Lung |
| DMS53 | 663 | Lung |
| SKMES1 | 787 | Lung |
| COR-L105 | 1520 | Lung |
| NCI-H1437 | 10000 | Lung |

**Supplemental Table 4: Baseline patient and disease characteristics by disease type**

| **Variable** | **Total cohort**  **(n = 40 patients)** | **Lower-risk MDS**  **(n = 17 patients)** | **Higher-risk MDS and MDS/MPN overlap (n = 16 patients)** | **AML**  **(n = 7 patients)** |
| --- | --- | --- | --- | --- |
| Age (median; range) | 74 years (46 – 84) | 74 years (67 – 81) | 74 years (59 - 84) | 66 years (46 – 80) |
| Male sex (n; %) | 27 (67.5%) | 11 (64.7%) | 11 (68.8%) | 5 (71.4%) |
| ECOG PS at baseline (n; %)  -- 0  -- 1  -- 2 | 4 (10.0%)  34 (85.0%)  2 (5.0%) | 3 (17.6%)  14 (82.4%)  0 | 1 (6.3%)  13 (81.3%)  2 (12.5%) | 0  7 (100%)  0 |
| Lines of prior therapy (n; %)  -- 0  -- 1  -- 2  -- ≥3 | 2 (5.0%)  7 (17.5%)  11 (27.5%)  20 (50.0%) | 1 (5.9%)  3 (17.6%)  4 (23.5%)  9 (52.9%) | 0  4 (25.0%)  6 (37.5%)  6 (37.5%) | 1 (14.3%)  0  1 (14.3%)  5 (71.4%) |
| Prior allogeneic hematopoietic stem cell transplant (n; %) | 4 (9.5%) | 1 (5.9%) | 0 | 3 (42.9%) |
| Baseline hemoglobin (median; range) | 8.45 g/dL (5.7 – 11.7) | 8.8 g/dL (5.7 – 11.7) | 8.1 g/dL (6.8 – 9.8) | 8.9 g/dL (7.5 – 10.9) |
| Baseline WBC (median; range) | 2.85 x 10^9^/L (0.8 – 32.6) | 3.4 x 10^9^/L (1.5 – 6.2) | 1.7 x 10^9^/L (0.8 – 32.6) | 2.0 x 10^9^/L (1.5 – 31.4) |
| Baseline platelets (median; range) | 54 x 10^9^/L (5 - 576) | 101 x 10^9^/L (9 – 413) | 48 x 10^9^/L (10 – 576) | 17 x 10^9^/L (5 - 142) |
| Baseline ANC (median; range) | 1.2 x 10^9^/L (0.1 – 18.7) | 1.5 x 10^9^/L (0.3 – 4.2) | 0.7 x 10^9^/L (0.1 – 18.7) | 0.6 x 10^9^/L (0.1 – 13.2) |
| IPSS-R (n; %)  -- very low  -- low  -- intermediate  -- high  -- very high  -- Unknown/not available | 1 (2.5%)  8 (20.0%)  10 (25.0%)  5 (12.5%)  7 (17.5%)  3 (7.5%) | 1 (5.9%)  8 (47.1%)  8 (47.1%)  0  0  0 | 0  0  1 (6.3%)  5 (31.3%)  7 (43.4%)  3 (18.8%) | N/A |
| ELN 2017 AML risk category (n; %)  -- favorable  -- intermediate  -- adverse  -- Unknown/not available | 0  1 (2.5%)  5 (12.5%)  1 (2.5%) | N/A | N/A | 0  1 (14.3%)  5 (71.4%)  1 (14.3%) |
| Baseline molecular abnormalities (n; %)  *-- SF3B1*  *-- SRSF2*  *-- U2AF1*  *-- ZRSR2*  *-- TP53*  *-- RUNX1*  *-- ASXL1*  *-- IDH1*  *-- IDH2*  *-- FLT3*  *-- NPM1*  *-- TET2*  *-- KRAS/NRAS*  *-- ETV6*  *-- DNMT3A* | 13 (32.5%)  8 (20.0%)  12 (30.0%)  2 (5.0%)  5 (12.5%)  4 (10.0%)  13 (32.5%)  2 (5.0%)  2 (5.0%)  1 (2.5%)  0  12 (30.0%)  9 (22.5%)  8 (20.0%)  4 (10.0%) | 9 (52.9%)  2 (11.8%)  3 (17.7%)  1 (5.9%)  0  1 (5.9%)  4 (23.5%)  1 (5.9%)  1 (5.9%)  0  0  6 (35.3%)  0  3 (17.7%)  3 (17.7%) | 2 (12.5%)  4 (25.0%)  6 (37.5%)  1 (6.3%)  4 (25.0%)  1 (6.3%)  7 (43.8%)  1 (6.3%)  0  1 (6.3%)  0  4 (25.0%)  6 (37.5%)  4 (25.0%)  1 (6.3%) | 2 (28.6%)  2 (28.6%)  3 (42.9%)  0  1 (14.3%)  2 (28.6%)  2 (28.6%)  0  1 (14.3%)  0  0  2 (28.6%)  3 (42.9%)  1 (14.3%)  0 |

**Supplemental Table 5: Baseline patient and disease characteristics by dose-escalation vs. dose-expansion cohort**

| **Variable** | **Dose-escalation cohort**  **(n = 11 patients)** | **Dose-expansion cohort**  **(n = 29 patients)** |
| --- | --- | --- |
| Age (median; range) | 75 years (68 – 84) | 73 years (46 – 81) |
| Male sex (n; %) | 9 (81.8%) | 18 (62.1%) |
| ECOG PS at baseline (n; %)  -- 0  -- 1  -- 2 | 0  10 (90.9%)  1 (9.1%) | 4 (13.8%)  24 (82.8%)  1 (3.4%) |
| Lines of prior therapy (n; %)  -- 0  -- 1  -- 2  -- ≥3 | 0  2 (18.2%)  5 (45.5%)  4 (36.4%) | 2 (6.9%)  5 (17.2%)  6 (20.7%)  16 (55.2%) |
| Disease  -- Lower-risk MDS  -- Higher-risk MDS  -- MDS/MPN overlap  -- AML | 5 (45.5%)  6 (54.5%)  0  0 | 12 (41.4%)  6 (20.7%)  4 (13.8%)  7 (24.1%) |
| Prior allogeneic hematopoietic stem cell transplant (n; %) | 1 (9.1%) | 3 (10.3%) |
| Baseline hemoglobin (median; range) | 8.0 g/dL (6.8 – 11.7) | 8.4 g/dL (5.7 – 10.9) |
| Baseline WBC (median; range) | 1.8 x 10^9^/L (0.2 – 12.7) | 3.1 x 10^9^/L (0.9 – 32.6) |
| Baseline platelets (median; range) | 52 x 10^9^/L (14 – 359) | 56 x 10^9^/L (5 – 576) |
| Baseline ANC (median; range) | 1.0 x 10^9^/L (0.1 – 3.2) | 1.5 x 10^9^/L (0.1 – 18.9) |
| IPSS-R (n; %)  -- very low  -- low  -- intermediate  -- high  -- very high  -- Unknown/not available | 1 (9.1%)  2 (18.2%)  2 (18.2%)  2 (18.2%)  4 (36.4%)  0 | 0  6 (27.3%)  7 (31.8%)  3 (13.6%)  3 (13.6%)  3 (13.6%) |
| ELN 2017 AML risk category (n; %)  -- favorable  -- intermediate  -- adverse  -- Unknown/not available | N/A | 0  1 (14.3%)  5 (71.4%)  1 (14.3%) |
| Baseline molecular abnormalities (n; %)  *-- SF3B1*  *-- SRSF2*  *-- U2AF1*  *-- ZRSR2*  *-- TP53*  *-- RUNX1*  *-- ASXL1*  *-- IDH1*  *-- IDH2*  *-- FLT3*  *-- NPM1*  *-- TET2*  *-- KRAS/NRAS*  *-- ETV6*  *-- DNMT3A* | 2 (18.2%)  3 (27.3%)  0  1 (9.1%)  2 (18.2%)  0  3 (27.3%)  1 (9.1%)  0  0  0  3 (27.3%)  1 (9.1%)  3 (27.3%)  1 (9.1%) | 11 (37.9%)  5 (17.2%)  12 (41.3%)  1 (3.4%)  3 (10.3%)  4 (13.8%)  10 (34.5%)  1 (3.4%)  2 (6.9%)  1 (3.4%)  0  9 (31.0%)  8 (27.6%)  5 (17.2%)  3 (10.3%) |

**Supplemental Table 6. Treatment-emergent adverse events (TEAE) related to PRT543.**

|  | **MDS (N=33) CTC Grade** | | | **AML (N=7) CTC Grade** | | | **Total (N=40) CTC Grade** | | |
| --- | --- | --- | --- | --- | --- | --- | --- | --- | --- |
| **Adverse event** | **1-2 n** | **3-5 n** | **Any n (%)** | **1-2 n** | **3-5 n** | **Any n (%)** | **1-2 n** | **3-5 n** | **Any n (%)** |
| **Number (%) of Subjects With Any Related TEAE** | **10** | **11** | **21 (62.9)** | **3** | **0** | **3 (42.9)** | **13** | **11** | **24 (60.0)** |
| **Hematologic TEAEs** |  |  |  |  |  |  |  |  |  |
| Anemia | 0 | 7 | 7 (21.2) | 0 | 0 | 0 | 0 | 7 | 7 (17.5) |
| Thrombocytopenia | 1 | 5 | 6 (18.2) | 0 | 0 | 0 | 1 | 5 | 6 (15.0) |
| Leukocytosis | 1 | 0 | 1 (3.0) | 0 | 0 | 0 | 1 | 0 | 1 (2.5) |
| Neutropenia | 0 | 1 | 1 (3.0) | 0 | 0 | 0 | 0 | 1 | 1 (2.5) |
| **Non-hematologic TEAEs** |  |  |  |  |  |  |  |  |  |
| Nausea | 6 | 0 | 6 (18.2) | 0 | 0 | 0 | 6 | 0 | 6 (15.0) |
| Diarrhea | 2 | 1 | 3 (9.1) | 1 | 0 | 1(14.3) | 3 | 1 | 4 (10.0) |
| Fatigue | 4 | 0 | 4 (11.4) | 0 | 0 | 0 | 4 | 0 | 4 (10.0) |
| Rash maculo-papular | 1 | 0 | 1 (3.0) | 1 | 0 | 1 (14.3) | 2 | 0 | 2 (5.0) |
| Decreased appetite | 1 | 0 | 1 (3.0) | 1 | 0 | 1 (14.3) | 2 | 0 | 2 (5.0) |
| Dyspnea | 2 | 0 | 2 (6.1) | 0 | 0 | 0 | 2 | 0 | 2 (5.0) |
| Constipation | 1 | 0 | 1 (3.0) | 0 | 0 | 0 | 1 | 0 | 1 (2.5) |
| Dry mouth | 0 | 0 | 0 | 1 | 0 | 1 (14.3) | 1 | 0 | 1 (2.5) |
| Gingival pain | 0 | 0 | 0 | 1 | 0 | 1 (14.3) | 1 | 0 | 1 (2.5) |
| Oral pain | 1 | 0 | 1 (3.0) | 0 | 0 | 0 | 1 | 0 | 1 (2.5) |
| Vomiting | 1 | 0 | 1 (3.0) | 0 | 0 | 0 | 1 | 0 | 1 (2.5) |
| Arthralgia | 1 | 0 | 1 (3.0) | 0 | 0 | 0 | 1 | 0 | 1 (2.5) |
| Bone pain | 1 | 0 | 1 (3.0) | 0 | 0 | 0 | 1 | 0 | 1 (2.5) |
| Muscle spasms | 1 | 0 | 1 (3.0) | 0 | 0 | 0 | 1 | 0 | 1 (2.5) |
| Muscular weakness | 1 | 0 | 1 (3.0) | 0 | 0 | 0 | 1 | 0 | 1 (2.5) |
| Pain in jaw | 0 | 0 | 0 | 1 | 0 | 1 (14.3) | 1 | 0 | 1 (2.5) |
| Chest discomfort | 1 | 0 | 1 (3.0) | 0 | 0 | 0 | 1 | 0 | 1 (2.5) |
| Dry skin | 0 | 0 | 0 | 1 | 0 | 1 (14.3) | 1 | 0 | 1 (2.5) |
| Eczema | 0 | 1 | 1 (3.0) | 0 | 0 | 0 | 0 | 1 | 1 (2.5) |
| Palmar-plantar erythrodysesthesia syndrome | 0 | 0 | 0 | 1 | 0 | 1 (14.3) | 1 | 0 | 1 (2.5) |
| Alanine aminotransferase increased | 1 | 0 | 1 (3.0) | 0 | 0 | 0 | 1 | 0 | 1 (2.5) |
| Aspartate aminotransferase increased | 0 | 0 | 0 | 1 | 0 | 1 (14.3) | 1 | 0 | 1 (2.5) |
| Blood creatinine increased | 0 | 0 | 0 | 1 | 0 | 1 (14.3) | 1 | 0 | 1 (2.5) |
| Weight decreased | 0 | 0 | 0 | 1 | 0 | 1 (14.3) | 1 | 0 | 1 (2.5) |
| Cognitive disorder | 1 | 0 | 1 (3.0) | 0 | 0 | 0 | 1 | 0 | 1 (2.5) |
| Dizziness | 1 | 0 | 1 (3.0) | 0 | 0 | 0 | 1 | 0 | 1 (2.5) |
| Dysgeusia | 0 | 0 | 0 | 1 | 0 | 1 (14.3) | 1 | 0 | 1 (2.5) |
| Headache | 0 | 0 | 0 | 1 | 0 | 1 (14.3) | 1 | 0 | 1 (2.5) |
| Epistaxis | 0 | 0 | 0 | 1 | 0 | 1 (14.3) | 1 | 0 | 1 (2.5) |
| Dry eye | 0 | 0 | 0 | 1 | 0 | 1 (14.3) | 1 | 0 | 1 (2.5) |
| Hyperbilirubinemia | 0 | 0 | 0 | 1 | 0 | 1 (14.3) | 1 | 0 | 1 (2.5) |
| Wound complication | 0 | 0 | 0 | 1 | 0 | 1 (14.3) | 1 | 0 | 1 (2.5) |

**Supplemental Table 7. Disease characteristics of responding patients.**

| **Patient** | **Disease** | **IPSS-R/ELN risk** | **Splicing mutation** | **Molecular** | **Response** |
| --- | --- | --- | --- | --- | --- |
| 9232-224-E3 | AML | Adverse | SRSF2 p.P95H | NRAS, ASXL1 | Cri; complete molecular response |
| 7130-104-B7 | HR-MDS | 7 (Very High) | SRSF2 p.P95L | ETV6, KRAS, PTPN11, SETBP1, ASXL1, ATR, TET2 | HI-E, HI-N, HI-P |
| 9117-193-E1 | MDS/MPN overlap | N/A | U2AF1 p.S34F | JAK2, ETV6, TERT, TET2 | HI-E |
| 7130-206-E1 | MDS/MPN overlap | 5.5 (high) | SF3B1 p.K700E | TET2 (2 variants), JAK2, TP53, EP300 | mCR |
| 4060-195-E3 | LR-MDS | 4 (intermediate) | U2AF1 p.Q157P | IDH2, ASXL1, RUNX1, ATRX, PHF6 | HI-E |
| 9297-191-E3 | AML | Adverse | SRSF2 p.P95H | TET2, NF1, RUNX1 (2 variants) | SD (platelet transfusion independence) |

AML – acute myeloid leukemia; CRi – complete remission with incomplete hematologic recovery; ELN – European LeukemiaNet; HI-E – hematologic improvement-erythroid; HI-N – hematologic improvement-neutrophil; HI-P – hematologic improvement-platelets; HR-MDS – higher-risk myelodysplastic syndrome; IPSS-R – international prognostic scoring system-revised; LR-MDS – lower-risk myelodysplastic syndrome; mCR – marrow complete remission; MDS/MPN – myelodysplastic syndrome/myeloproliferative neoplasm; SD – stable disease

**Supplemental Table 8. Baseline characteristics of patients included in RNA splicing analyses.**

| **Patient** | **Disease** | **IPSS-R/ELN risk** | **Splicing mutation** | **Molecular** | **Response** |
| --- | --- | --- | --- | --- | --- |
| 9232-180-E3 | LR-MDS | 3 (low) | SRSF2 p.P95H | TET2 (2 variants), MPL, ATM | SD |
| 9297-191-E3 | AML | Adverse | SRSF2 p.P95H | TET2, NF1, RUNX1 (2 variants) | SD (platelet transfusion independence) |
| 9222-198-E3 | HR-MDS | 7 (very high) | SRSF2 p.P95H | ASXL1, STAG2, LRP1B, KIT, ATM, NRAS, ARID1B, EP300 | SD |
| 9236-216-E3 | HR-MDS | 5 (high) | U2AF1 p.Q157P | NRAS, SETBP1, ASXL1, MPL, CSF3R, JAK2 | PD |
| 4080-245-E3 | AML | Intermediate | U2AF1 p.Q157P | IDH2, ASXL1, RUNX1, ATRX, PHF6 | SD |

AML – acute myeloid leukemia; ELN – European LeukemiaNet; HR-MDS – higher-risk myelodysplastic syndrome; IPSS-R – international prognostic scoring system-revised; LR-MDS – lower-risk myelodysplastic syndrome; PD – progressive disease; SD – stable disease

**Supplemental Table 9: List of IRBs**

| **Site** | **PI** | **IRB Type** | **IRB Address** |
| --- | --- | --- | --- |
| **Master** | Master | IntegReview/Advarra | IntegReview  3815 S. Capital of Texas Hwy, Suite 320 Austin, TX 78704  Tel. 512.326.3001  Local Fax. 512.697.0085  [http://www.integreview.com](http://www.integreview.com/)  Later bought out by: Advarra IRB  6100 Merriweather Dr., Suite 600  Columbia, MD 21044  410-884-2900 |
| **3000** | TONash (McKean) | IntegReview/Advarra | IntegReview  3815 S. Capital of Texas Hwy, Suite 320 Austin, TX 78704  Tel. 512.326.3001  Local Fax. 512.697.0085  [http://www.integreview.com](http://www.integreview.com/)  Later bought out by: Advarra IRB  6100 Merriweather Dr., Suite 600  Columbia, MD 21044  410-884-2900 |
| **4060** | FCS Sarasota (Patel) | IntegReview/Advarra | IntegReview  3815 S. Capital of Texas Hwy, Suite 320 Austin, TX 78704  Tel. 512.326.3001  Local Fax. 512.697.0085  [http://www.integreview.com](http://www.integreview.com/)  Later bought out by: Advarra IRB  6100 Merriweather Dr., Suite 600  Columbia, MD 21044  410-884-2900 |

| **Site** | **PI** | **IRB Type** | **IRB Address** |
| --- | --- | --- | --- |
| **4080** | FCS Lake Mary (Jauhari) | IntegReview/Advarra | IntegReview  3815 S. Capital of Texas Hwy, Suite 320 Austin, TX 78704  Tel. 512.326.3001  Local Fax. 512.697.0085  [http://www.integreview.com](http://www.integreview.com/) Later bought out by:  Advarra IRB  6100 Merriweather Dr., Suite 600  Columbia, MD 21044  410-884-2900 |
| **7102** | Atlantic Health (Cherry) | Local IRB | Atlantic Health Systen 100 Madison Avenue  Morristown, NJ 07960 |
| **7122** | UPMC (Zandberg) | IntegReview/Advarra (as a local IRB) | IntegReview  3815 S. Capital of Texas Hwy, Suite 320 Austin, TX 78704  Tel. 512.326.3001  Local Fax. 512.697.0085  [http://www.integreview.com](http://www.integreview.com/) Later bought out by:  Advarra IRB  6100 Merriweather Dr., Suite 600  Columbia, MD 21044  410-884-2900 |
| **7130** | Norton (Stevens) | WIRB/WCG | WIRB  1019 39th Ave. SE, Suite 12  Puyallup, WA 98374-2115  360-252-2500 |
| **9021** | Augusta U (Cortes) | WIRB/WCG | WIRB  1019 39th Ave. SE, Suite 12  Puyallup, WA 98374-2115  360-252-2500 |
| **9055** | Ochsner (Finn) | Local IRB | Ochsner Clinic Foundation Institutional Review Board  1514 Jefferson Highway New Orleans, LA 70121 |
| **9117** | MDACC  (Verstovsek) | Local | MD Anderson  Office of Human Subject Protection 7007 Bertner Avenue – Unit 1637  Houston, TX 77030 |
| **9117B** | MDAnderson (Ferrarotto) | Local | MD Anderson  Office of Human Subject Protection 7007 Bertner Avenue – Unit 1637  Houston, TX 77030 |

| **Site** | **PI** | **IRB Type** | **IRB Address** |
| --- | --- | --- | --- |
| **9121** | U of Michigan (Swiecicki) | Local | University of Michigan  Medical School Institutional Review Board 2800 Plymouth Road, Building 520, Suite  3214  Ann Arbor, MI 48109 |
| **9122** | U of WA (Rodriguez) | WIRB/WCG | WIRB  1019 39th Ave. SE, Suite 12  Puyallup, WA 98374-2115  360-252-2500 |
| **9152** | COH (Khaled) | WIRB/WCG | WIRB  1019 39th Ave. SE, Suite 12  Puyallup, WA 98374-2115  360-252-2500 |
| **9160** | OSU (Baiocchi) | WIRB/WCG | WIRB  1019 39th Ave. SE, Suite 12  Puyallup, WA 98374-2115  360-252-2500 |
| **9163** | Christiana Care (Guarino) | Local | Christiana Care Institutional Review Board  Helen F. Graham Cancer Center & Research Institute  West Pavilion - Suite 2350 4701 Ogletown-Stanton Road  Newark, Delaware 19713 |
| **9166** | Montefiore (Acuna-Villaorduna) | Brany | Brany  1981 Marcus Avenue, Suite 210 Lake Success, NY 11042 |
| **9185** | UCSF (Kang) | Local | University of California San Francisco Human Research Protection Program, Box 1288  Institutional Review Board 490 Illinois Street, Floor 6 San Francisco, CA 94158 |
| **9203** | DFCI (Garcia) | Local | Dana Farber Cancer Institute Office for Human Research Studies 450 Brookline Avenue, BP332A Boston, MA 02215 |
| **9222** | U of Iowa (Monga) | Local | University of Iowa  Human Subjects Office/ Institutional Review Board  105 Hardin Library for the Health Sciences 600 Newton Road  Iowa City, Iowa 52242 |
| **9232** | Moffitt (Kuykendall) | Advarra | Advarra IRB  6100 Merriweather Dr., Suite 600  Columbia, MD 21044  410-884-2900 |

| **Site** | **PI** | **IRB Type** | **IRB Address** |
| --- | --- | --- | --- |
| **9297** | Banner Health (Nath) | IntegReview/Advarra | IntegReview  3815 S. Capital of Texas Hwy, Suite 320 Austin, TX 78704  Tel. 512.326.3001  Local Fax. 512.697.0085  [http://www.integreview.com](http://www.integreview.com/) Later bought out by:  Advarra IRB  6100 Merriweather Dr., Suite 600  Columbia, MD 21044  410-884-2900 |
| **9304** | Sidney Kimmel (Keiffer) | IntegReview/Advarra | IntegReview  3815 S. Capital of Texas Hwy, Suite 320 Austin, TX 78704  Tel. 512.326.3001  Local Fax. 512.697.0085  [http://www.integreview.com](http://www.integreview.com/) Later bought out by:  Advarra IRB  6100 Merriweather Dr., Suite 600  Columbia, MD 21044  410-884-2900 |
| **9305** | Levine/Atrium (Chojecki) | Advarra | Advarra IRB  6100 Merriweather Dr., Suite 600  Columbia, MD 21044  410-884-2900 |

**Supplemental Figure legends:**

**Supplemental Figure 1. PRT543 is a potent and selective PRMT5 inhibitor.** **(A)** Chemical structure of PRT543. The co-crystal structure of the PRMT5/MEP50 complex with PRT543 was solved at resolution of 2.5 Å. The electron density of PRT543 in the SAM binding pocket was well defined and exhibited similar interactions in other PRMT5/MEP50 structures containing SAM/SAH analogs. **(B)** Mode of binding of PRT543 was assessed by plotting IC_50_ values against the concentration of SAM (express as [SAM]/K_M, SAM_), and substrate peptide (express as [substrate]/ K_M, peptide_). **(C)** Jump dilution assay showing PRMT5/MEP50 enzymatic progress curve in the absence and presence of PRT543 (n = 2). **(D)** Concentration-dependent inhibition of PRMT5/MEP50 enzymatic activity by PRT543 in a scintillation proximity based radiometric assay. Data represent mean ± SD. n = 11. **(E)** Biochemical selectivity of PRT543 against 37 human methyltransferases. Percent control represents % enzymatic activity remaining in the presence of 10 μM PRT543 relative to DMSO. (**F)** Dose-dependent reduction of symmetrically dimethylated SMD3 following 3 days of PRT543 treatment. **(G)** Profile of anti-proliferative response to PRT543 in a panel of 85 cell lines following 10 days of treatment.

**Supplemental Figure 2. Anti-tumor activity of PRT543 in vivo***.* Oral daily administration of PRT543 leads to dose-dependent tumor growth inhibition in the HEL92.1.7 and SET2 xenograft models.

**Supplemental Figure 3. Baseline cytogenetic and molecular characteristics and association with response**. Baseline cytogenetic and molecular characteristics of patients treated with PRT543 stratified by disease type (AML vs MDS and MDS/MPN) and responses are shown. Response was defined as CR, CRi, mCR, PR, MLFS, and HI per ELN 2017 AML and IWG 2006 MDS response criteria.

**Supplemental Figure 4. Maximum change in variant allele fraction (VAF) of splicing mutations.** The maximum change of the VAF of splicing mutations from baseline for individual patients is shown as a waterfall plot. The type of splicing mutation is color-coded. AML – acute myeloid leukemia; CRi – complete remission with incomplete hematologic recovery; HI – hematologic improvement; mCR – marrow complete remission; MDS – myelodysplastic syndrome; MDS/MPN – myelodysplastic syndrome/myeloproliferative neoplasm; NE – not evaluable; PD – progressive disease; SD – stable disease

**Supplemental Figure 5. Aberrant splicing events induced by PRT543**. **(A)** RNA-seq coverage plots at specific gene loci where significant intron retention events have been detected on PRT543 vs. DMSO/pre-treatment (yellow). The tracks colored in gray represent control samples (cell lines treated with DMSO or patient samples pre-treatment), while the tracks colored in red represent samples treated with PRT543. The reads are adjusted by the maximum value parameter (y-axis) which controls for sequencing depths of the samples.**(B)** Venn diagram of significant differential alternative splicing events in PRT543 vs. DMSO-treated in K562 cells with wildtype and distinct splicing factor mutations. **(C)** Gene set enrichment analysis of the 347 common events found in all cell lines after PRT543 treatment using the human MSigDB hallmark gene sets*.* **(D)** Clustering of all cell lines and patient samples based on gene expression from RNA-seq. **(E)** Heatmap showing differential gene expression of all available pre- and post-treatment treatment patient samples that underwent RNA-seq. The pre- and post-treatment samples from the same patient clustered together. **(F)** Gene set enrichment analysis of all differentially expressed genes post-treatment vs. pre-treatment in patient samples using the human MSigDB hallmark gene sets.

**References:**

1. Shen S, Park JW, Huang J, et al. MATS: a Bayesian framework for flexible detection of differential alternative splicing from RNA-Seq data. *Nucleic Acids Res*. 2012;40(8):e61.

2. Shen S, Park JW, Lu ZX, et al. rMATS: robust and flexible detection of differential alternative splicing from replicate RNA-Seq data. *Proc Natl Acad Sci U S A*. 2014;111(51):E5593-5601.

3. Park JW, Tokheim C, Shen S, Xing Y. Identifying differential alternative splicing events from RNA sequencing data using RNASeq-MATS. *Methods Mol Biol*. 2013;1038:171-179.

4. Chen EY, Tan CM, Kou Y, et al. Enrichr: interactive and collaborative HTML5 gene list enrichment analysis tool. *BMC Bioinformatics*. 2013;14:128.

5. Kuleshov MV, Jones MR, Rouillard AD, et al. Enrichr: a comprehensive gene set enrichment analysis web server 2016 update. *Nucleic Acids Res*. 2016;44(W1):W90-97.

6. Xie Z, Bailey A, Kuleshov MV, et al. Gene Set Knowledge Discovery with Enrichr. *Curr Protoc*. 2021;1(3):e90.

7. Huber W, Carey VJ, Gentleman R, et al. Orchestrating high-throughput genomic analysis with Bioconductor. *Nat Methods*. 2015;12(2):115-121.

8. Liao Y, Smyth GK, Shi W. featureCounts: an efficient general purpose program for assigning sequence reads to genomic features. *Bioinformatics*. 2014;30(7):923-930.

9. Law CW, Chen Y, Shi W, Smyth GK. voom: precision weights unlock linear model analysis tools for RNA-seq read counts. *Genome Biol*. 2014;15(2):R29.

10. Love MI, Huber W, Anders S. Moderated estimation of fold change and dispersion for RNA-seq data with DESeq2. *Genome Biol*. 2014;15(12):550.
