# Supplementary figures and images for: Phase Ib study of PRT543, an oral protein arginine methyltransferase 5 (PRMT5) inhibitor, in patients with advanced splicing factor-mutant myeloid malignancies
Source: Leukemia. 2025 Jan 24;39(3):765–9. doi: 10.1038/s41375-025-02515-8 (PMC11879867; doi:10.1038/s41375-025-02515-8)

## Slide 1
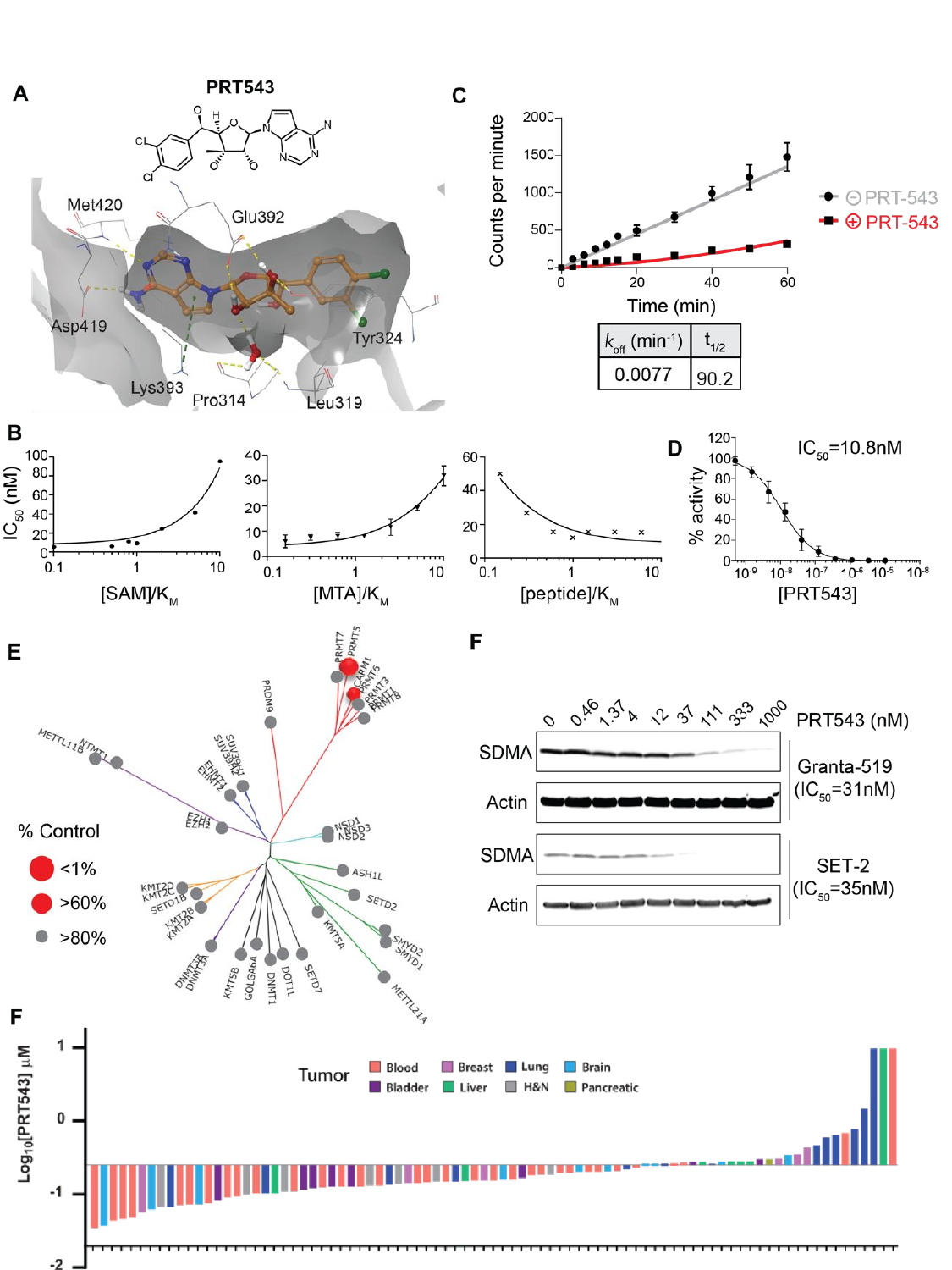

Supplement: Supplementary file 2 — Supplemental Figure 1. PRT543 is a potent and selective PRMT5 inhibitor. [file 41375_2025_2515_MOESM2_ESM.pptx]

## Slide 1
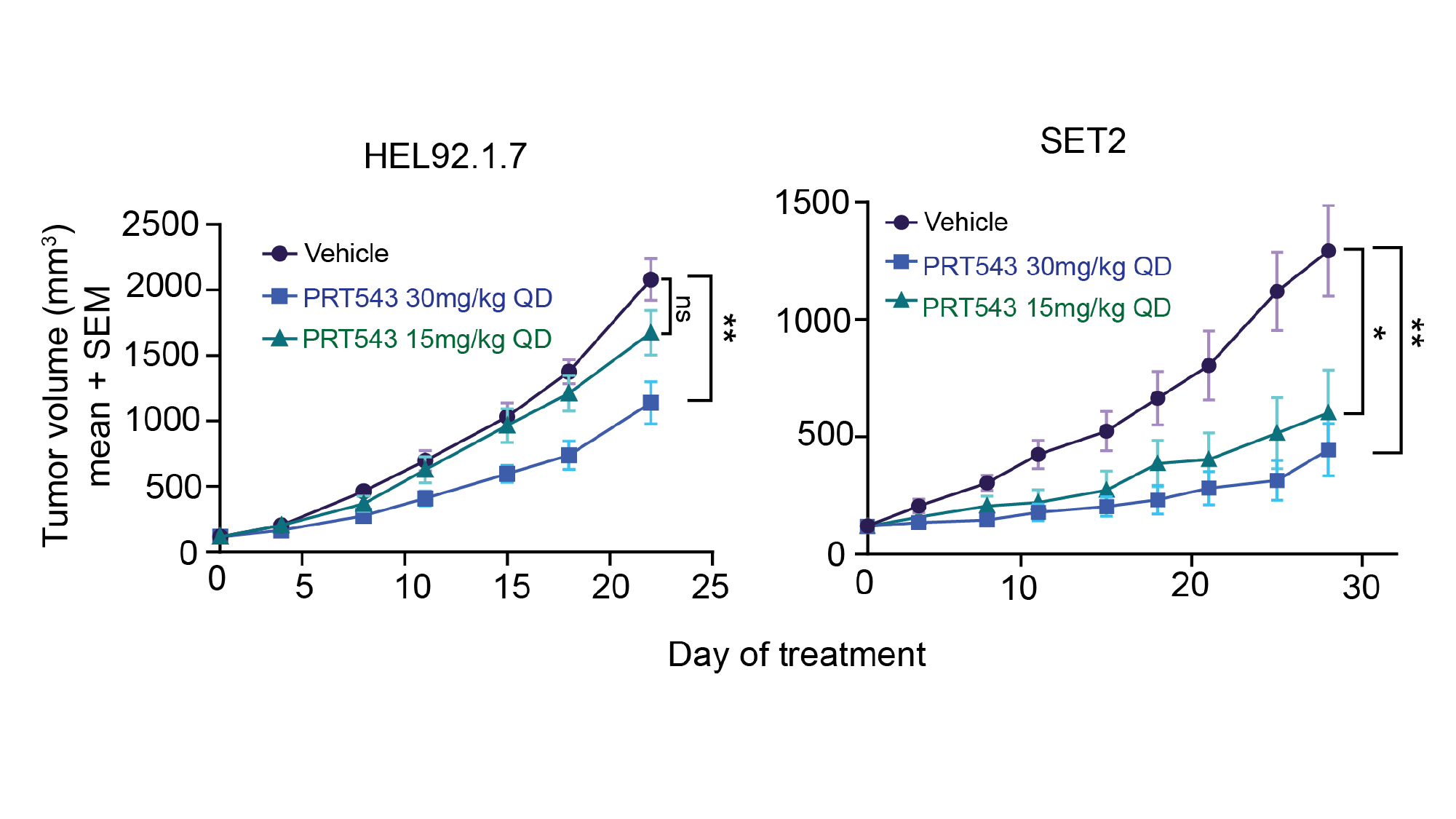

Supplement: Supplementary file 3 — Supplemental Figure 2. Anti-tumor activity of PRT543 in vivo. [file 41375_2025_2515_MOESM3_ESM.pptx]

## Slide 1
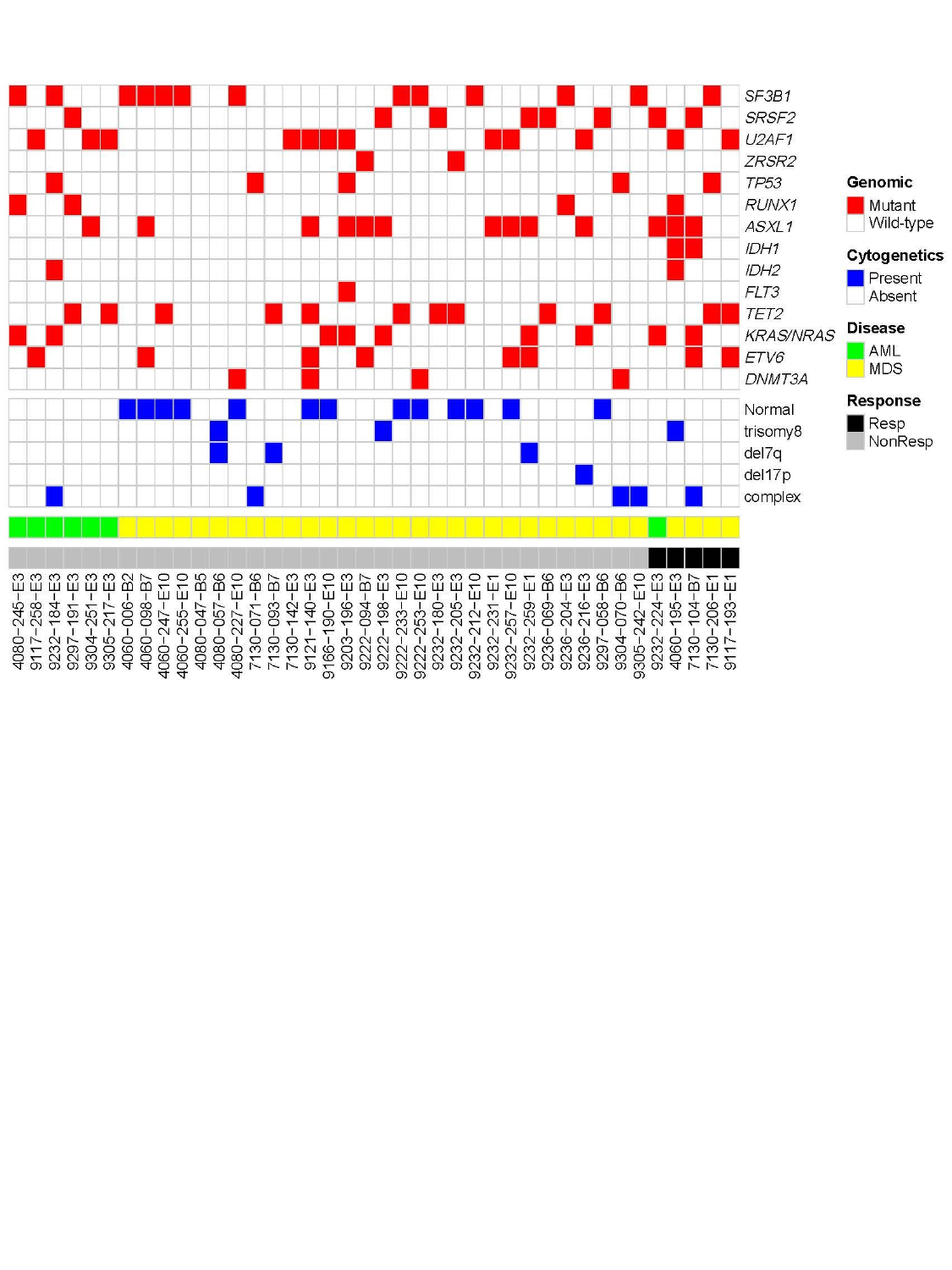

Supplement: Supplementary file 4 — Supplemental Figure 3. Baseline cytogenetic and molecular characteristics and association with response. [file 41375_2025_2515_MOESM4_ESM.pptx]

## Slide 1
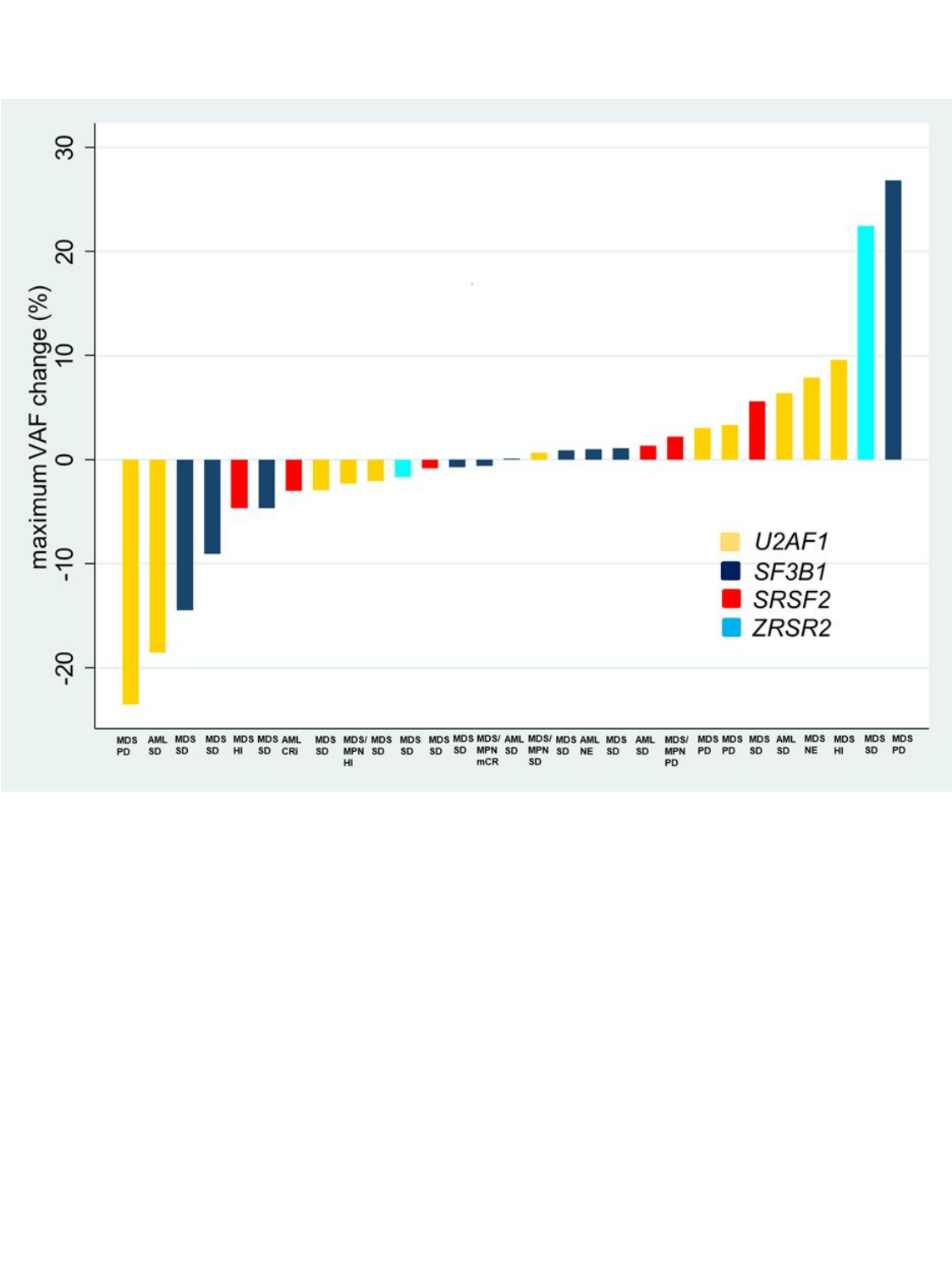

Supplement: Supplementary file 5 — Supplemental Figure 4. Maximum change in variant allele fraction (VAF) of splicing mutations. [file 41375_2025_2515_MOESM5_ESM.pptx]

## Slide 1
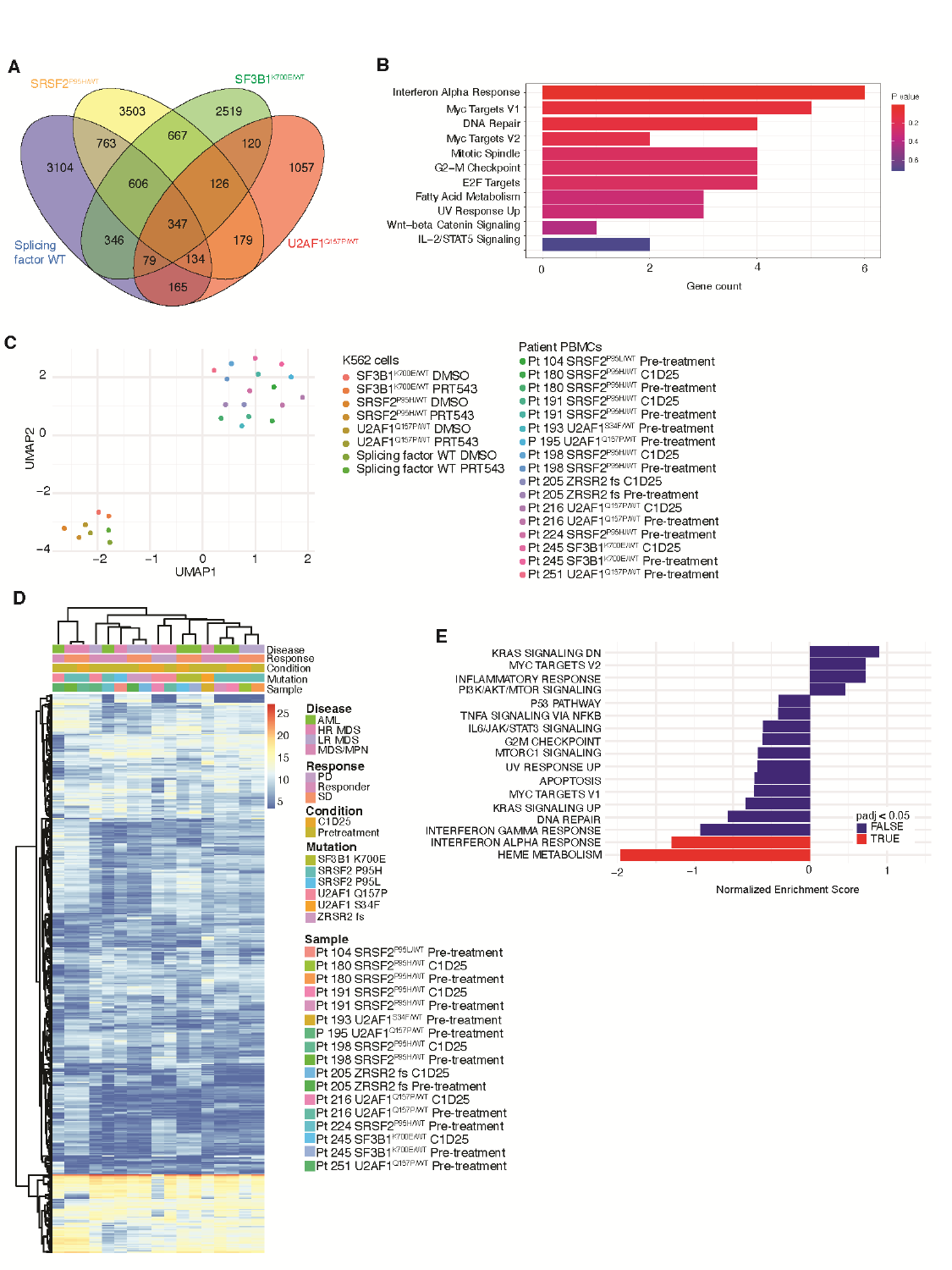

Supplement: Supplementary file 6 — Supplemental Figure 5. Aberrant splicing events induced by PRT543. [file 41375_2025_2515_MOESM6_ESM.pptx]
